# Clinical Mass Spectrometry in the Bioinformatics Era: A Hitchhiker’s Guide

**DOI:** 10.1016/j.csbj.2018.08.003

**Published:** 2018-08-28

**Authors:** Yeow-Kuan Chong, Chi-Chun Ho, Shui-Yee Leung, Susanna K.P. Lau, Patrick C.Y. Woo

**Affiliations:** a Hospital Authority Toxicology Reference Laboratory, Department of Pathology, Princess Margaret Hospital (PMH), Kowloon, Hong Kong; b Chemical Pathology and Medical Genetics, Department of Pathology, Princess Margaret Hospital (PMH), Kowloon, Hong Kong; c Division of Chemical Pathology, Department of Clinical Pathology, Pamela Youde Nethersole Eastern Hospital (PYNEH), Hong Kong; d Division of Clinical Biochemistry, Department of Pathology, Queen Mary Hospital (QMH), Hong Kong; e Centre for Genomic Sciences, The University of Hong Kong, Hong Kong; f Department of Ocean Science, School of Science, The Hong Kong University of Science and Technology, Kowloon, Hong Kong; g Department of Microbiology, Li Ka Shing Faculty of Medicine, The University of Hong Kong, Hong Kong; h State Key Laboratory of Emerging Infectious Diseases, Department of Microbiology, The University of Hong Kong, Hong Kong; i Research Centre of Infection and Immunology, The University of Hong Kong, Hong Kong; j Carol Yu Centre for Infection, The University of Hong Kong, Hong Kong; k Collaborative Innovation Center for Diagnosis and Treatment of Infectious Diseases, The University of Hong Kong, Hong Kong

## Abstract

Mass spectrometry (MS) is a sensitive, specific and versatile analytical technique in the clinical laboratory that has recently undergone rapid development. From initial use in metabolic profiling, it has matured into applications including clinical toxicology assays, target hormone and metabolite quantitation, and more recently, rapid microbial identification and antimicrobial resistance detection by matrix assisted laser desorption ionization-time of flight mass spectrometry (MALDI-TOF MS). In this mini-review, we first succinctly outline the basics of clinical mass spectrometry. Examples of hard ionization (electron ionization) and soft ionization (electrospray ionization, MALDI) are presented to demonstrate their clinical applications. Next, a conceptual discourse on mass selection and determination is presented: quadrupole mass filter, time-of-flight mass spectrometer and the Orbitrap; and MS/MS (tandem-in-space, tandem-in-time and data acquisition), illustrated with clinical examples. Current applications in (1) bacterial and fungal identification, antimicrobial susceptibility testing and phylogenetic classification, (2) general unknown urine toxicology screening and expanded new-born metabolic screening and (3) clinical metabolic profiling by gas chromatography are outlined. Finally, major limitations of MS-based techniques, including the technical challenges of matrix effect and isobaric interference; and novel challenges in the post-genomic era, such as protein molecular variants, are critically discussed from the perspective of service laboratories. Computer technology and structural biology have played important roles in the maturation of this field. MS-based techniques have the potential to replace current analytical techniques, and existing expertise and instrument will undergo rapid evolution. Significant automation and adaptation to regulatory requirements are underway. Mass spectrometry is unleashing its potentials in clinical laboratories.

## 1 Introduction

Mass spectrometry (MS) is an analytical technique which measures the mass-to-charge ratio of ions generated from analytes [1], allowing the qualitative identification and quantitative determination of analytes. First put into functional forms by physicist Joseph J. Thomson (1856 – 1940) and chemist Francis W. Aston (1877 - 1945) [[2], [3], [4]], biochemical researchers and clinical scientists were relatively late adopters of the technique. In fact, when MS was first adopted by clinical laboratorians in the early 1970s, it was only an investigative tool for metabolic profiling in urine [[5], [6], [7], [8]] and other body fluids [[9], [10], [11]]. The first clinical laboratory applications of MS were toxicology assays targeting therapeutic drugs, drugs of abuse and their metabolites [[12], [13], [14], [15], [16], [17]]. With the rapid development of immunoassays in the 1970 to 1980s, first with radioactive labelling [[18], [19], [20]], then with enzymes [21] and fluorophores [22]; and the paradigm shift from phenotyping towards genotyping in the 1990s, largely facilitated by PCR [23], automated DNA sequencing technologies [24] and human and microbial genome projects [25,26], the many virtues of MS-based techniques were outshone. Coupled to the often prohibitive instrument cost, requirement for interpretative expertise and lack of substantial automation in many MS-based techniques, the application of MS in the clinical laboratory remained limited and, with due respect, often regarded as a reference or verification technique, for drugs, hormones [[27], [28], [29]] and one of the chemotaxonomic techniques for establishing microbial phylogeny [30,31].

While many of these applications remain pertinent to today’s clinical laboratory - as detailed in the selected examples here – we, as practicing pathologists, have also been witnessing a disruptive change that is being brought about by MS-based techniques. We see manual or semi-automatic phenotyping of microorganisms vanish into the literal dark and hollow *Tunnel of TOF* and emerge as literal identities with quality scores from computer screens (MS-based microbial identification). We see the rainbow colours from a battery of tests, hinting at vague structural similarities among sugars, ketones or smaller organic molecules and inorganic ions, evaporate into *chromatographs* and condense as countless invisible mass spectra capable of enlightening the minimal structural differences. We see the glorious fleet of immunoassay analysers, intricately engineered and armed with multiple pipettes, reaction vessels and spectrophotometers to quantify analytes from small molecules of drugs to large and heterogenous glycopeptides, slowly tarnish as MS-based methods for specific, multi-target quantitation silently creep from research institutions to service-based laboratories.

In this review, we first outline the hardware and configurations of mass spectrometry, namely (1) ion source and ionization, (2) mass selection and determination and, perhaps of cardinal importance in current applications, (3) tandem mass spectrometry. Next, we illustrate the clinical laboratory application of each of the common MS configurations with specific examples in microbial identification, toxicology and clinical metabolomics, which are all made possible by the advances in bioinformatics in the past decades. Due to the wide-ranging characteristics of samples arising from the diverse applications, from crude body fluid and bacterial colonies to organic solvent extracts and purified peptides, the importance – or the lack thereof – of sample pre-treatment and analyte purification is also highlighted for the examples discussed. Finally, as researchers, end-users and advocates, we nevertheless critically discuss the known weaknesses and pitfalls with MS-based methods, which have sometimes led to incongruences with alternate contemporary modalities and offer some of our views and certain possible solutions.

## 2 Basics of Mass Spectrometry

### 2.1 The ABCs of MS: The *Hards* and *Softs* of Ionization

Mass spectrometry procedures begin with the generation of ions by different ionization techniques [1]. Ionization techniques can be classified into soft ionization techniques and hard ionization techniques, based on the amount of energy imparted to the analyte [32]. These techniques first opened the door to the study of small molecules, and subsequently biological macromolecules. The importance of soft ionisation methods, such as electrospray ionization (ESI) and matrix-assisted laser desorption/ionization (MALDI), for the analysis of these macromolecules is engraved in the 2002 Nobel Prize in Chemistry, awarded jointly to John Fenn (1917 - 2010) and Tanaka Koichi (1959 - ) [[33], [34], [35], [36]]. As can be seen in the sections that follow, ESI and MALDI underlie important clinical laboratory applications such as toxicological screening and microbial identification.

Hard ionization refers to techniques with which a high amount of energy is imparted upon an analyte [37], causing disruption of covalent bonds. A classic example is electron ionization (EI) [38], in which the molecules of interest are fragmented into a number of “daughter ions” of various sizes; the most extreme example, however, would be inductively coupled plasma mass spectrometry (ICP-MS), in which analytes are subject to the extreme heat of an argon plasma, resulting in the formation of chiefly monoatomic ions [39]. Soft ionization, in contrast, refers to techniques with which the covalent bonds in an analyte are not disturbed. Soft ionization techniques used in the clinical laboratory would include EI [40], atmospheric pressure chemical ionization (APCI) [41,42], chemical ionization (CI) [43], and MALDI [36]. As applications progressed, EI (in GC-MS), ESI (with liquid chromatography tandem mass spectrometry [LC-MS/MS]) and MALDI (coupled with TOF), emerged as the three most important ionization techniques in the modern clinical laboratory.

#### 2.1.1 Electron Ionization

**Fig. 1:**
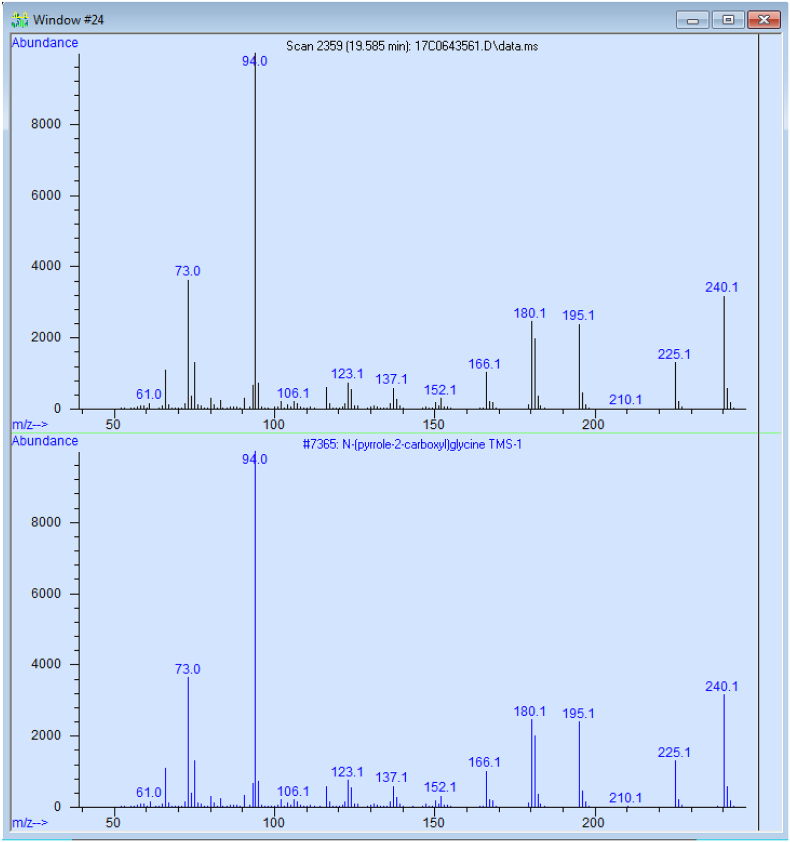
Spectral-matching for identification of rare disease markers. The use of probability-based matching together with the highly reproducible EI spectra of compounds stored in spectral libraries enable the clinical laboratory to identify abnormal metabolites present in a specimen without the need to obtain chemical standards in advance. Depicted in above, N-(pyrrole-2-carboxyl) glycine, a marker of hyperprolinaemia type II (OMIM: # 239510), is found in a sample received for urine metabolic screening in the one of the authors’ laboratory.

EI was developed, according to declassified research after the Second World War, in the 1930-40s [44,45] and it assumed dominating importance in MS until the widespread availability of the soft ionization techniques and tandem mass spectrometry. EI involves heating of a metal filament to produce thermionic electrons, which are subsequently accelerated by a potential difference. These highly energized electrons collide on a stream of target molecules separated by a chromatographic system, commonly a gas chromatograph, and cause simultaneous fragmentation and ionization of targets [46]. The most significant benefit of using EI in clinical MS analysis is its reproducibility across different instruments, even those from different manufacturers: by accelerating bombarding electrons to a standard energy of 70 eV, fragmentation of target molecules is consistent enough for comparable mass-spectra libraries to be compiled and shared via the Internet [47]. It allows the tentative identification of a disease marker, even though the analysing laboratory may not have procured the chemical standard (“the positive control”) in question (Fig. 1). The interpretation of EI mass spectra is beyond the scope of this review and readers are referred to the excellent text by McLafferty and Turecek [48].

#### 2.1.2 Electrospray Ionization

Electrospray ionization (ESI) is a soft ionization technique. It is built upon the basic technique of electrospray, in which a solution is pumped through a capillary, on which a high potential is applied. This results in the formation of a cone-shaped fluid surface, the Taylor cone, from which droplets are emitted [49]. Although not entirely accurate, the process is analogous to electrostatic spray painting, in which the paint (the solution containing the analyte) is turned into charged and very fine droplets or even dry powder (“ionized”), traverse a distance, and eventually deposit onto the metal object to be coated, such as car body panels (the detector in the mass spectrometer). Depending on the potential applied on the capillary, these droplets in ESI would contain an excess of either positive or negative charge [50]. As solvent evaporates from the droplets, electrostatic repulsion of excess charge would exceed surface tension of the droplet (Rayleigh limit), leading to Coulomb fission – essentially an “explosion”, creating many more fine droplets [51]. In a modern ESI ion source, heating and nitrogen gas are used to improve the efficiency of ion formation and transmission by evaporating the solvent [52]. While a higher temperature during the ESI process logically leads to better solvent evaporation and better availability of gaseous phase ion into the mass spectrometer, enhancing the sensitivity of the experiment, the efficiency gain is limited by increased diffusion and loss of analyte from collision and thermodegradation of analytes [53].

With the ability to form ions from molecules dissolved in a solution, ESI is commonly coupled with liquid chromatography [40]. It allows formation of both positive and negative ions [51]. The ability of the ESI to generate multiply-charged ions allowed the analysis of macromolecules by moving the mass-to-charge ratio within the reach of the mass analyser [54,55]. The ionic species formed in ESI are generally protonated molecular ion in the positive mode and deprotonated molecular ion in the negative mode [1]; adducts, including ammonium and sodium adducts in positive mode, and chloride and formate adducts in negative mode, may result from presence of ionic species in the solvent used [[56], [57], [58]]. The efficiency of ESI is well known to be affected by the flow rate of the eluent: the lower the flow rate, the higher the efficiency of electrospray ionization [59]. The improved efficiency at extremely low flow rate is best demonstrated in the coupling of ESI with nano-liquid chromatography and micro-liquid chromatography [60].

#### 2.1.3 Matrix-Assisted Laser Desorption Ionization

Matrix-assisted laser desorption ionization (MALDI) was developed by Karas and Kruger [61] and applied to the analysis of macromolecules by Tanaka [36]. In contrast to EI and ESI, which primarily work with gas and liquid, respectively, MALDI works with solid analytes: samples are usually mixed with a UV-absorbing matrix solution and dried to obtain a crystalized matrix [62], and pulsed laser is then applied to the analyte-embedded matrix, leading to ionization of the analyte [63].

A two-step framework for the mechanism of MALDI has been proposed: the primary ionization step where ions are first formed during the laser pulse and the secondary reactions that follow in the desorption/ablation plume, with the first step occurring in the nanosecond timescale and the secondary reaction in the microsecond timescale [64]. While multiple models have been proposed for the ion formation in MALDI in recent years, the mechanism itself remains controversial [[65], [66], [67], [68]].

#### 2.1.4 Emerging Ionization Techniques

Apart from the traditional techniques, ambient ionization techniques such as direct analysis in real time (DART) which is based on atmospheric pressure chemical ionization [69], and desorption electrospray ionization (DESI) which is based on electrospray ionization [70], have been thoroughly investigated as research tools [71,72]. They are not commonly used in clinical laboratories, except in tertiary centres where non-biological specimens are examined [73].

### 2.2 Until Detection: Ions Fly, Far and High

Before mass detectors eventually detect the abundance of ions and their mass-to-charge ratio, mass analysers are responsible for filtering and separation of the incoming ions. Additionally, selected ions may be further fragmented or trapped in certain mass analyser configurations. Common mass analysers used in the clinical laboratory include the quadrupole, time-of-flight, and Orbitrap mass analysers [74,75]. Other types of mass analyser, such as magnetic sector and Fourier-transform ion cyclotron resonance (FT-ICR), are less commonly used [76,77]. Mass analyser designs differ by their range of mass-to-charge ratio that could be measured, whether they can operate in pulse or continuous mode, the linear dynamic range of ion intensity that could be measured and their mass resolution and accuracy [1,37].

#### 2.2.1 The Quadrupole

**Fig. 2:**
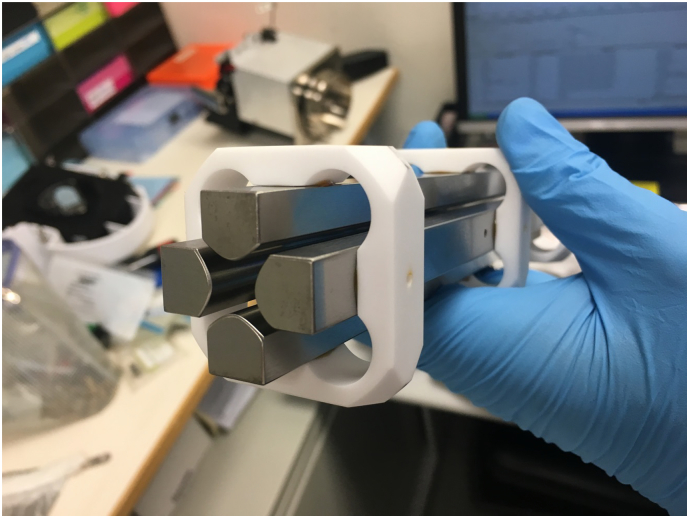
The quadrupole. Quadrupole mass filter assembly of an Agilent 6430 LC-MS/MS instrument (Agilent, Santa Clara, CA, USA). The inner hyperbolic surface is important in the formation of electrical fields. Screw-holes on the metallic rods are where they become connected to the electrical supply.

The quadrupole mass analyser (Fig. 2) comprises two pairs of rods that are positioned parallel to each other in a radial array, arranged in a hyperbolic or circular cross section, with opposing rods being electrically connected [78,79]. The quadrupole mass filter operates by generating an electrical field, inside which only ions within a set range of mass-to-charge ratio may pass through; this is achieved through the application of radiofrequency (RF) and direct current (DC) signals across the two pairs of opposing rods, forming a low-pass filter and a high-pass filter in two orthogonal planes [78]. The stability of ions inside the electrical field of a quadrupole mass filter can be calculated by solving the Mathieu equation [80]: the higher the DC signal applied with respect to the RF signal, the more selective is the mass filter towards the mass-to-charge ratio of the ions that pass through, but at the same time, the less absolute amount of ions of the selected mass-to-charge-ratio would pass [81]. In the case when no DC signal is applied, the quadrupole allows all ions to pass through with minimal loss [82], as can be seen with the collision cell in triple quadrupole mass spectrometers [83] and the collision/ reaction cell used in inductively-coupled plasma mass spectrometry [84]. The quadrupole mass filter is traditionally considered a low resolution instrument as it usually operates at unit resolution, though higher resolutions have been used in an attempt to achieve better specificity [85].

Quadrupole mass filters operate in a continuous manner [81], and is therefore useful in combination with chromatographic techniques and ion sources (e.g. electron ionization and electrospray ionization) that operates in a continuous manner. Quadrupole mass filters are rugged and inexpensive, and therefore are widely used in the clinical laboratory in conjunction with gas chromatography as GC-MS [86] and in liquid chromatography as either triple quadrupole or in combination with time-of-flight or Orbitrap mass analysers [87].

#### 2.2.2 Time-of-Flight

**Fig. 3:**
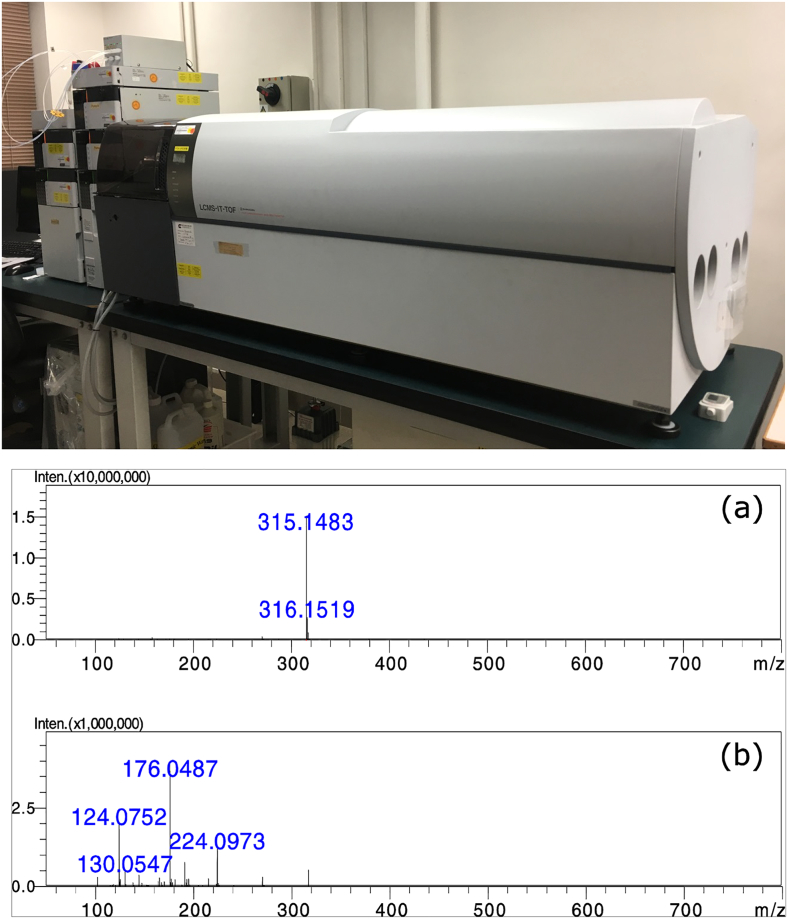
Mass spectrometry for structural elucidation. Upper panel: LCMS-IT-TOF mass spectrometer (Shimadzu, Kyoto, Japan) in one of the authors’ laboratory (PMH). The combination of a quadrupole ion trap (IT) with a time-of-flight mass analyser facilitates the structural elucidation of drug analogues by allowing MS^n^ measurement with high resolution and accuracy. Lower panel: (a) MS1 spectrum of ranitidine (molecular formula, C_13_H_22_N_4_O_3_S; monoisotopic mass 314.141 g/mol), showing the protonated molecular ion (315.1483 Da). (b) MS2 spectrum of ranitidine showing major fragments (176.0487 Da, 124.0752 Da and 224.0973 Da), allowing confirmation of the identity of the compound.

A TOF mass analyser to ions is analogous to a chromatographic column in traditional chemical separation to compounds with varying physical and chemical properties. A TOF mass analyser accelerates ions through an electric field, and, as the ions travel through the flight tube, they become separated based on their velocity – which, in turn, is determined by the charge they acquired at the ion source and their mass. As the voltage from the ion source (the energy given) and length of the flight tube (the travelling distance) are kept constant, the mass-to-charge ratio of the analysed molecule becomes proportional to the time-of-flight squared and can be easily determined [88]. The time-of-flight mass spectrometer has a theoretically unlimited mass range [89] and therefore is suited to the analysis of macromolecules, particularly if coupled with ESI and MALDI techniques [90]. A time-of-flight mass spectrometer is shown in Fig. 3.

Unlike the quadrupole, a TOF-MS operates in a pulsed manner and is therefore more suited to pulsed ionization techniques such as MALDI, where pulsed laser light is used to create ions [91]. Compared to Quadrupole instruments that have a resolving power of 1,000 to 10,000 FWHM, modern TOF-MS are able to achieve mass resolving power up to 50,000 FWHM (“full width at half maximum”, which can be visualized as how far one can “zoom-in” to a mass peak) [[92], [93], [94]]. It is expected that ultrafast electronics will enable TOF mass analysers to achieve even higher resolutions [95,96]. In contrast to the ruggedness of quadrupole mass filters, TOF mass analysers are sensitive to temperature changes due to thermal expansion of the flight tube. It also requires a high vacuum, and has a narrower dynamic range for quantitation [95].

#### 2.2.3 Ion Traps

Ion traps are devices which can be used to store gaseous ions, and some designs of ion traps can be used in mass spectrometers, such as the quadrupole ion trap (QIT) which has been widely used [97,98]. A QIT consists of three shaped electrodes (two end-cap electrodes and one ring electrode) which, together form an enclosed volume within which ions can be confined by a radiofrequency signal applied [99]. Another type of ion trap, the linear ion trap (LIT), is discussed below in the context of tandem mass spectrometry.

The QIT operates in a pulsed mode: ions packets following injection are stopped by collision with a buffer gas such as helium and are confined [99,100]. Manipulation of ions such as selective ejection and tandem mass spectrometry (collision-induced dissociation with the buffer gas) can be performed by changing the radiofrequency signal applied. This enables multistage tandem mass spectrometry (MS^n^), which is a major benefit of using a quadrupole ion trap mass spectrometer [100].

The adoption of ion trap mass spectrometers has not been widespread in the clinical laboratory: first, these instruments have a limited dynamic range of quantitation due to the space charge effect (confined charge density becomes too high and mass accuracy and resolution become negatively affected) [101]; second, while an ion trap instrument is able to perform tandem mass spectrometry, daughter ions with mass-to-charge ratios lower than approximately one third of the parent ions cannot be identified with tandem mass spectrometry in a quadrupole ion trap (due to the difficulties in containing low m/z ions and fragmenting the parent ion at the same time, resulting in the so-called “1/3 rule” or low mass cut off effect) [102]. Hybrid ion trap-time-of-flight instrument has been developed to provide the ability to perform MS^n^ experiments with the benefit of high resolution mass spectrometry [103].

#### 2.2.4 Orbitrap

**Fig. 4:**
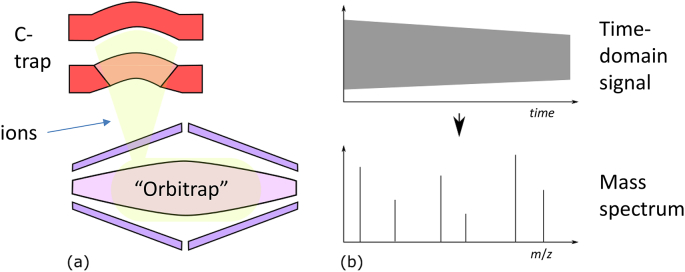
Spectral acquisition by Orbitrap and C-trap. (a) During spectral acquisition, the C-trap (in red) focuses and inject ions into the orbitrap mass analyser (in purple). (b) The time domain signal obtained in (a) is converted into a mass spectrum by the mathematical process of Fourier transformation.

An Orbitrap® ion trap mass analyser/ detector comprises three electrodes: a central electrode and two cup-shaped outer electrodes (Fig. 4). In contrast to the mass analysers discussed above, ion detection in Orbitrap® is by the measurement of image current generated by the circular movement of ions around the central electrode so the Orbitrap® is also a mass detector [104,105]. The frequency of the signal is related to the inverse square root of the mass to charge ratio of the ions, and the image current generated is the sum total of signal generated by all the ions trapped in the Orbitrap® [106], and the ion abundance and mass to charge ratio is detected as the magnitude and frequency of signal after Fourier transformation from the time domain signal to a frequency domain signal akin to that in Fourier transform ion cyclotron resonance mass spectrometry [107]. The C-trap, used in most orbitrap analyser nowadays, is an RF-only quadrupole that forms a C-shaped arc which allows the orbitrap to couple with any ion source, quadrupoles, or linear ion traps [108].

Orbitrap® operates in a pulsed manner and is therefore suited to pulsed ionization techniques. Modern orbitrap analyser is able to achieve resolving power of over 200,000 FWHM [109]. The extremely long flight path, sometimes up to hundreds of kilometres, necessitates an extremely high vacuum of 2 × 10^-10^ mbar [105]. This leads to a correspondingly high cost of acquisition in comparison to TOF-based mass spectrometers, though the recent introduction of entry-level models enabled its competition with high-end TOF analysers [110].

### 2.3 Thou Shalt Fly Again: MS in Tandem

Regardless of configuration, the goal of MS/MS is to break down parent ions into fragment daughter ions, which can be profiled in detail (e.g. for structural confirmation), or selectively quantified (e.g. for quantitative assays). The prototypical depiction of MS/MS is the triple quadrupole MS/MS, with two quadrupole mass analysers and a collision cell situated in between [111]. Collision-induced dissociation (CID) is one of the major methods in the production of ion fragments in MS/MS used in the clinical laboratory [112]. The principle behind CID is inelastic collision: precursor ions, accelerated by a potential, is exposed to a neutral inert gas (e.g. nitrogen) in the collision cell, which result in conversion of kinetic energy into internal energy, and with resultant fragmentation of the precursor ions [113]. In addition to the collision energy, the pressure inside the collision cell affects the characteristic of the spectra: at higher pressure, the probability of multiple collisions increases, and this results in breaking of higher-energy bonds, as well as more extensive breakdown of precursor ions [114].

The triple quadrupole tandem mass spectrometer (TQ-MS) described above is an example of tandem-in-space mass spectrometer. In a TQ-MS, each of the two quadrupole mass analysers (termed “Q1” and “Q3”) can operate in one of the three modes (Table 1). The operation modes of a TQ-MS are by operating the first and second quadrupole analysers in combination (Table 2). Multiple reaction monitoring is the mode most commonly used in the clinical laboratory: it allows quantification of analytes by integrating the ion response for a carefully-chosen pair of precursor-product ions [115], and importantly, confirmation of identity of the compound by the ratio of peak area between precursor-product ion pairs. The product ion scan is the second most important mode used in the clinical laboratory: in product ion scan mode, fragment ions are identified and this mode is used to help select precursor-product ion pairs used in multiple reaction monitoring [116]. Product ion scan is also helpful in confirming the identity of a compound, and comparison of product ion scan results may allow tentative identification of drugs [117]. The other modes, i.e. neutral loss (detection of the loss of a specific neutral fragment) and precursor scan (detection of the loss of a specific charged fragment), have been used for profiling of acylcarnitines [118] and identification of drug-glucuronide conjugates [119,120].

**Table 1:** Operation modes of a quadrupole mass analyser.

Operation mode	Description
RF-only	No selection of mass-to-charge ratio occurs; all ions are passed through.
Scan	Ions of different mass-to-charge ratios are passed at different time.
Select	Ions of one particular mass-to-charge ratio are allowed to pass through.

**Table 2:** Operation modes of a triple quadrupole tandem mass spectrometer.

Operation mode	Q1	Q3
Product ion scan	Select	Scan
Precursor ion scan	Scan	Select
Neutral loss	Scan	Scan
Multiple reaction monitoring	Select	Select

## 3 Clinical Mass Spectrometry

### 3.1 Clinical Applications: I. Microbial Identification, Speciation, and Beyond

While the MALDI-TOF MS based identification of pathogens has been extensively reported and reviewed, whether generally in comparison to traditional phenotypic and biochemical methods, or specifically to narrow or broad groups of pathogens of interest such as streptococci [121], anaerobic bacteria [122], fungi [123] or even parasites [124]; it is obligatory to highlight, if only very succinctly, the principle and practice of the procedure.

#### 3.1.1 Practical Aspects of MALDI-TOF MS Microbial Identification

**Fig. 5:**
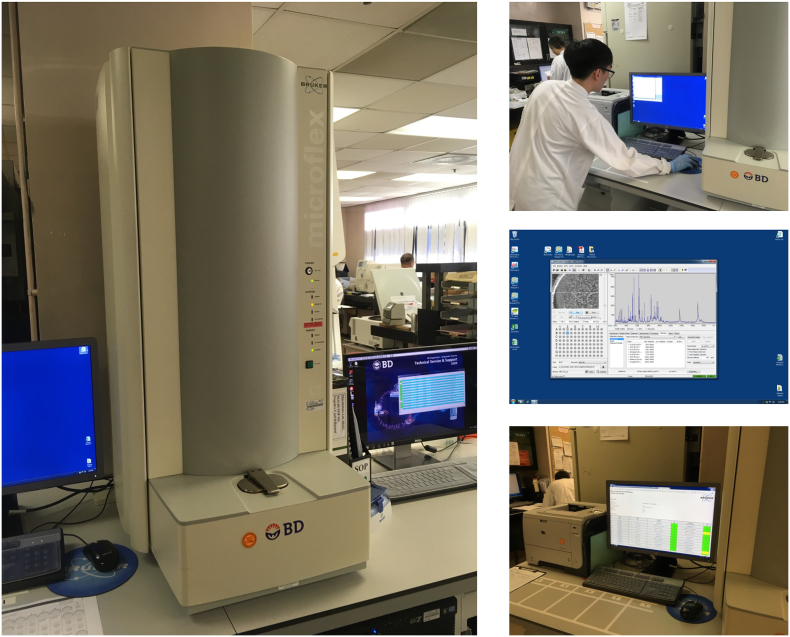
Microbial identification by MALDI-TOF MS in the clinical laboratory. Shown in the left, the MALDI-TOF MS-based MALDI Biotyper® system in use in the authors’ laboratory (PYNEH). The whole process requires minimal hands-on time for the operator, and the spectral acquisition operation is fully computer-controlled (top right). Spectral data and other monitoring parameters are displayed in real-time during the spectral acquisition process for quality assurance and system diagnostics purposes (middle right). Finally, automatic spectral matching and identification is performed by the software accompanying the instrument, outputting best match organism identity and an objective score value (bottom right).

**Fig. 6:**
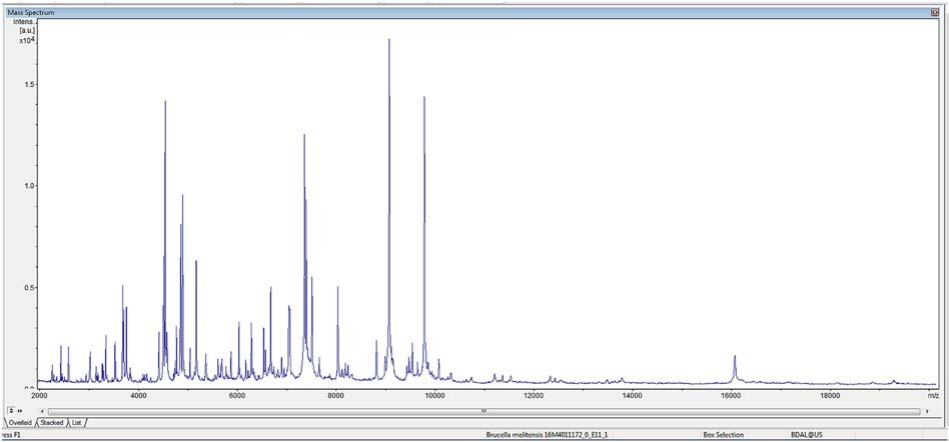
Rapid identification of potential bioterrorism agents in the clinical laboratory. The spectral display of a *Brucella melitensis* isolate, a biosafety level 3 (BSL-3) and Category B bioterrorism agent classified by the US Center for Disease Control and Prevention (CDC). Showing the acquired mass spectra range from 2,000 to 20,000 *m*/*z*, which corresponds to a mass range of 2 to 20 kDa as most of the ions produced by MALDI are of charge 1^+^. It should be noted that, unlike chemical identification, the exact protein or peptide fragment identity of the *m/z* peaks is often not known; usually, only a database search of standard spectra from definitively identified organisms is performed and no attempt is made to identify individual fragments. This seemingly simply approach nevertheless allows the early and rapid identification of microorganisms, hence facilitate clinical management and epidemiological investigation. The MALDI-TOF biotyping method also reduces the potential risks to the laboratory professional as the matrix solvent often inactivates the organism (see text), and that the minimal handling steps further reduces aerosolization and infective risks.

MALDI-TOF MS based microbial identification (Fig. 5) is typified by the identification of cultured single colony of bacteria or fungi. First, the bacterium is preferably freshly cultured or sub-cultured on a solid medium, such as a blood agar plate. After an overnight growth, a representative colony is picked with a sterile, disposable tool, such as a pipette tip, plastic loop or toothpick, and smeared on designated areas of a steel target plate supplied by the instrument manufacturer [125]. The smear is then overlaid with the suggested matrix solution, which can either be a mixture of (a) alpha-cyano-4-hydroxy-cinnamic acid, acetonitrile and trifluoroacetic acid (for the US FDA-approved Bruker or bioMérieux systems) or (b) 2,5-dihydroxybenzoic acid, water, ethanol and acetonitrile (for the Shimadzu system, not available in the USA) [126]. For bacteria with particular biosafety concerns, such as the biosafety level 3 (BSL-3) agents *Burkholderia pseudomallei*, *Burkholderia mallei* and *Brucella* species (Fig. 6), pre-treatment by the matrix solvent [127] or solvent components [128] can enable their safe handling in accordance with biosafety level 2 (BSL-2) practices [129]. After the matrix solution is air-dried, the target plate is loaded onto the integrated MALDI-TOF MS instrument for automatic mass spectra generation. The spectra are then matched against a database of reference spectra, usually provided by the manufacturer but may also be expanded by the laboratory by using spectra from custom isolates [130]. A matching score is usually produced by the automatic software accompanying the instrument, which consistently compares the peaks from the mass spectra of the clinical isolate, known spectra in the library and the correlation of intensities thereof. This technology reduces the time needed to definitively identify an unknown bacterial isolate to the species level form about 24 - 36 hours in routine laboratories (e.g. by rapid or conventional biochemical tests) to about 5 minutes [126], with accuracy approaching 16S rRNA gene sequencing and additional savings in manpower and reagent costs. Special protocols are available for the processing of microbes with thick cell walls or spore-forming organisms with biosafety concerns to ensure proper protein extraction or inactivation for safe handling [128,[131], [132], [133]]. Although MALDI-TOF MS is well-established to be comparable or superior to conventional biochemical tests in terms of accuracy, we are obliged to emphasize that it is still impossible to completely replace DNA sequencing of conserved regions (e.g. 16S rRNA gene sequencing for bacteria and ITS sequencing for fungi) in reference laboratories, due to its current database limitations [[134], [135], [136], [137], [138]] and inability to confirm novel microbial species [[139], [140], [141], [142]].

While clinical microbiology laboratories have embraced MS-based technology only in the past decade or so, with the potentials of rapid identification and susceptibility testing of mirco-organisms and substantial input from the instrument vendors, MALDI-TOF MS bacterial identification systems, following only HPLC-MS/MS newborn screening and tacrolimus assay [143], are among the first actual MS-based diagnostic devices to obtain FDA 510(k) clearance and serve as direct replacement of current laboratory test procedures [144]. In contrast, despite relatively early adoption of the analytical technique in fields such as toxicology and inborn errors of metabolism, the guidance for clinical use of mass spectrometry has been minimal until the Clinical and Laboratory Standards Institute (CLSI) published the CLSI C62-A document in late 2014 [145].

Practically, when compared to other traditional techniques, microbial identification by MALDI-TOF MS offers a number of major advantages. In terms of efficiency, the current commercially available platforms are easy to use and require significantly reduced technical manpower in the clinical laboratory compared to sequential staining and biochemical phenotyping. This also potentially translates to an improved turnaround time, earlier identification results and better clinical care [146]. In terms of effectiveness, the biological and phylogenetic basis of MALDI-TOF identification, as discussed below, enables the routine clinical laboratory to deliver reference-standard identification, occasionally rivalling the accuracy of definitive gene sequencing techniques [147] – although database expansions and optimizations remain a priority [148,149]. Last but not least, the great potentials of MALDI-TOF in clinical microbiology also lies in its ability to perform an “extended phenotyping”, including the detection of antimicrobial resistance and toxin production, which is also further elaborated.

#### 3.1.2 The Theoretical Basis of MALDI-TOF MS Microbial Identification

We can justifiably view MALDI-TOF MS microbial identification as a happy marriage between phenotypic and genetic methods. In the pre-genomic era, microbiologists have already made use of tests such as fatty acid profiling to define bacterial species and species groups, and the proteomic-based study of microorganisms has also led to better understanding and sometimes improved methods in classifying bacteria. Nevertheless, the “stable” genotype – in contrast to phenotypic features that can often vary depending on growth conditions – still form the basis of gold standard and reference methods such as DNA-DNA hybridization [150], ribosomal RNA gene sequencing [151] and multi-locus sequence typing [152]. By measuring the mass spectra produced by microbial proteins ranging from about 2,000 to 20,000 Da, the MALDI-TOF method captures a proteomic signature comprising all major proteins (Fig. 6), in particularly, the ribosomal proteins that are highly expressed [153] and enriched by the extraction protocols [154]. This feature is of phylogenetic importance: in a large-scale study published by researchers at the National Center for Biotechnology Information (NCBI), it was found that horizontal gene transfer (HGT) is rampant among the prokaryotes and even involved some of the traditional phylogenetic marker genes such as *groEL* [[155], [156], [157]], whereas no HGT event was found in the study for any of the ribosomal proteins [158]. If phylogenetic methods, such as maximum parsimony, maximum likelihood [159,160] or Bayesian methods [161] with appropriate priors can be applied to the analysis of MALDI-TOF ribosomal protein signatures, this widespread clinical method will hold promise to a new generation of low-cost, high-resolution microbial phylogeny – perhaps for the first time in microbiology, initiating not from reference laboratories or research centres, but rather from the network of service laboratories routinely using the MALDI-TOF MS technology and cumulating as novel proposals for bacterial and fungal classification and species discovery.

#### 3.1.3 Emerging Applications of MALDI-TOF MS in Clinical Microbiology

Since the early days of MALDI-TOF MS, approaches to utilize the technology for antibiotic resistance have been proposed. They can be broadly divided into optimizations of the analyser and added steps to the protocol to detect (1) antibiotics and their potential degradation products, (2) microbial proteins or even non-protein components of the pathogen that are associated with resistance, and (3) genetic and genomic signatures of resistance [162]. Semi-quantitative growth rate analysis using MALDI-TOF MS for documenting resistance has also been proposed [163]; it can be considered a molecular reinstatement of the microscopic observation drug susceptibility assay (MODS), initially developed for use in primitive settings in Peru [164]. Of these methods, the “functional assay” approach to detect antibiotic degradation products has seen most promise in resistance detection [165,166] and has been integrated into software packages provided by the MALDI-TOF MS vendors [167]. As for the detection of characteristic microbial proteins, it has been applied in the subtyping of clinically important groups that are otherwise not fully resolved by their routine proteomic mass spectra, such as KPC carbapenemase-producing *Klebsiella pneumoniae* [168], methicillin-resistant *Staphylococcus aureus* producing the phenol-soluble modulin toxin [169] and *Bacteroides fragilis* strains producing the metallo-beta-lactamase CfiA [170]. It should perhaps be pointed out, that although antibiotic “metabolite” detection (from the antibiotic hydrolysis assay supernatant) is certainly possible with a MALDI-TOF MS analyser, it is only an flexible adaptation of the instrument, and that other MS configurations described in this clinical review, such as LC-MS [171,172], may be equally fit for purpose. In terms of antibiotic/ antibiotic metabolite assays, an LC-MS system intended for drug screening is likely to be more suitable than a MALDI-TOF MS station initially designed for rapid bacterial identification [173].

#### 3.1.4 The Future of MALDI-TOF MS in Clinical Microbiology

We can expect the continued development of MALDI-TOF MS to eventually achieve a consistent, broadly-applicable and efficient biotyping method which can at least parallel traditional phenotyping at all levels: provided that the phenotype basis of original classification is mediated by a protein or cellular component amenable to, and is above the limit of detection of the MS instrument. While certain limitations in identification, such as that for closely-related species groups including *Burkholderia pseudomallei* and *Burkholderia thailandensis* [129]; *Streptococcus mitis* / *oralis* and *S. pneumonia* [174]; and *Tsukamurella* species [175], have previously been reported, it is worth noting that these “blind spots” are mostly due to inadequate database coverage and do not represent a weakness of the technique *per se*. As for other “problem areas” of MALDI-TOF MS, such as the often-quoted inability to differentiate between *Escherichia coli* and *Shigella* species, they merely represent the initial emphasis of the method in using phylogenetically-informative proteins (such as the ribosomal proteins); whole-genome and pan-genome studies have repeatedly demonstrated that the two genera, while each harbour specific genetic features [176,177], are not phylogenetically distinct [178,179]. The use of a representative range of isolates to establish a MALDI-TOF MS proteomic signature, and more flexible, non-linear classification algorithms (such as artificial neural networks), have led to successful application of the technology to biotype such non-monophyletic groups [180]. In the near future, with improvements in species- or strain-specific marker identification [181], spectral signal processing technology and enhanced sensitivity of MALDI-TOF MS instruments, this versatile phenotypic identification tool can potentially be more widely applied in microbial identification in mixed or contaminated culture [182], sensitive detection from culture broth [183] and even direct identification of pathogens from blood and other body fluids without the need of bacterial or fungal culture [184,185].

### 3.2 Clinical Applications: II. Toxicology, Targeted Detection, Screening and Discovery

LC-MS, commonly used as LC-TOF-MS and LC-MS/MS, has seen much growth in its clinical use in the last two decades. LC-MS have been applied in applications such as urine toxicology screening and endocrine testing of glucocorticoids [186], mineralocorticoids [187,188], biogenic amines and metanephrines [189] and sex steroids [190,191]. Though no chromatography is used, newborn screening utilizing MS/MS is nevertheless an important application of MS/MS and has certain operational similarities to other MS/MS techniques in the clinical laboratory [192]. These applications often reflect the combined advantage LC-MS over alternative techniques: higher specificity, especially when compared to immunoassays for low molecular weight compounds such as drugs [193] and steroidal hormones [190,191]; higher sensitivity, which often obviates the need for elaborate sample concentration in photometric techniques such as those by diode array detection; and, last but not least, without the need for an extra derivatization step to enhance analyte volatility and thermo-stability as in GC-MS.

#### 3.2.1 Urine Toxicology Screening

General unknown toxicology screening in urine is a prime example to illustrate the strength of mass spectrometry coupled to liquid chromatography. The separation power of LC is combined with the sensitivity and specificity of mass spectrometry; the result is simplification of sample preparation and increase in the spectrum of detection of analytes, compared with predecessor techniques [194]. The aim of general unknown toxicology screening is to allow identification of drugs and toxins in biological fluid without a priori knowledge as to the nature of such drugs and toxins. LC coupled with TOF mass spectrometry is considered the technique of choice for urine toxicology screening [195].

**Fig. 7:**
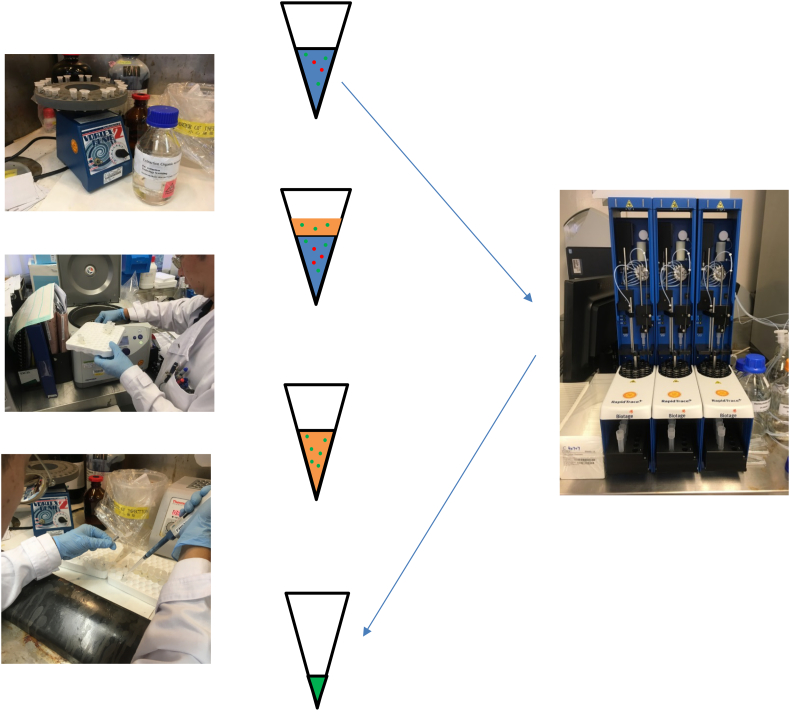
The then and now of sample preparation. Traditionally, sample preparation for LC-MS analysis require pre-treatment steps to remove interfering substances (red) dissolved in the original matrix (blue), particularly those that can participate in matrix-effect to create ion-suppression and enhancement, and to concentrate the analytes of interest (green) in a usually organic solvent (orange). These processes typically involve solvent extraction (top left), centrifugation (middle left), multiple pipetting steps (bottom left) and eventual drying under an inert atmosphere (not shown). Modern sample preparation is facilitated by automated, cartridge-based solid-phase extraction (right) with enhanced throughput and consistency.

Traditionally, toxicology analysis is done after sample preparation with solid-phase [[196], [197], [198]] or liquid-liquid extraction [199], followed by reconstitution and analysis (Fig. 7). With modern TOF mass analysers, sensitivity gain allow injection of diluted urine without prior extraction [200]. Injection is followed by reverse phase chromatography and ESI-TOF mass spectrometry operating in positive mode. Most currently-deployed TOF mass spectrometers do not allow for quick polarity-switching; as such, as most common drugs and toxins are best detected with positive ESI, many laboratory would opt for one run with positive ionization [195], reserving further analysis if the specimen requires analysis for analytes such as frusemide and hydrochlorothiazide [201], as well as valproic acid [202] which are detected with negative ESI.

The use of TOF mass spectrometry provides two layers of specificity on top of chromatographic separation: first, high resolution mass spectrometry combined with isotopic distribution allows determination of elemental composition (i.e. the molecular formula) of the compound of interest [203]; and secondly, the use of in-source fragmentation by operating the ESI at high aperture voltage allows tandem-in-time mass spectrometry and detection of product ions with high mass accuracy [195].

#### 3.2.2 Expanded Newborn Screening: Chromatography-Free MS/MS in the Clinical Laboratory

It has been more than two decades since tandem mass spectrometry is first used for the diagnosis of inborn errors of metabolism [[204], [205], [206]]. Following the reports of the New South Wales Newborn Screening Program [207] and the New England Newborn Screening Program [208], expanded newborn screening by MS/MS have been implemented worldwide in over 30 countries [209].

Expanded newborn screening by MS/MS generally involve the analysis of amino acids and acylcarnitines in dried blood spots, which allows the diagnosis of amino acidopathies, organic acidurias, as well as fatty acid oxidation defects [207,208]; additional markers may be included based on the incidence of specific metabolic disorders in the locality [209,210].

Dried blood spots are collected by depositing a few drops of whole blood onto filter paper, nowadays often put into a card format. Chemically, filter paper used in dried blood spots sampling are either cotton-based or glass microfibre-based [211], and solvent extraction from dried blood spots can be seen as a form of supported liquid extraction [212]. In comparison with biological fluids commonly assayed in the clinical laboratory, dried blood spots are stable at room temperature and can be transported/mailed in regular envelopes [211,213]; and the small volume requirement (30-70 μL of whole blood is required per blood spot) made it possible to use even lancet-prick samples [214], which minimizes pain and discomfort for the patient compared to a full-volume blood draw.

The analytical procedure begins with extraction of analytes from the dried blood spot with addition of internal standards, followed by injection into a liquid chromatography-tandem mass spectrometer, operating without a chromatography column in flow-injection mode [215]. Electrospray ionization together with tandem mass spectrometry operating in multiple reaction mode are typically used [[216], [217], [218]]. In the authors’ laboratory, dried blood spot analysis is done on specimens collected on Whatman 903 filter paper, using a commercial assay kit (NeoBase Non-derivatized MSMS, Perkin Elmer, MA) with which semi-quantitation of amino acids and acylcarnitines are performed with deuterated internal standards [219].

The use of tandem mass spectrometry without liquid chromatography in expanded newborn screening allows analysis of samples in a few minutes [218]; the downside of this is that isobaric compounds cannot be distinguished [204] and subsequent testing by LC-MS/MS and GC-MS are necessary after identification of metabolic diseases [220,221].

### 3.3 Clinical Applications: III. Metabolomics, Metabolites and Disease Markers

GC-MS has long been used in the clinical laboratory: its use for clinical diagnosis can be traced to the 1970s, with the early focus being on poisoning [222] and inborn errors of metabolism such as organic aciduria [223,224] and disorders of steroidogenesis [225,226]. It is the basis of definitive and reference methods for many analytes including cholesterol [227], creatinine [228], and cortisol [229]. While it is no longer possible to say that GC-MS is “the most widely used application” [74] of mass spectrometry in the clinical laboratory, the superior separation and non-selective nature of detection explains the continuing use of GC-MS in urine steroid profiling [230,231], metabolic screening [232,233], and toxicology [234].

**Fig. 8:**
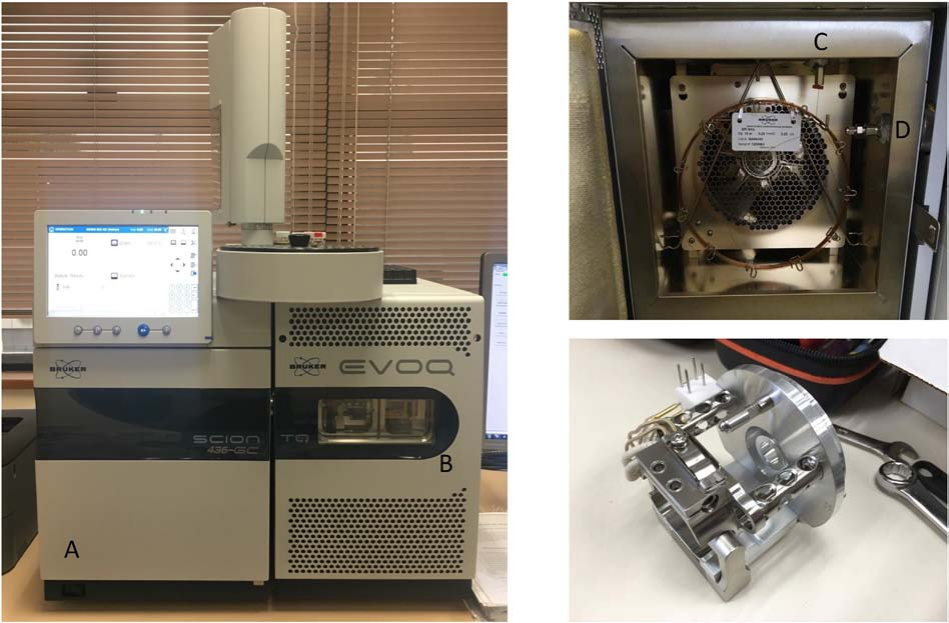
Miniaturization in clinical metabolomics. A Bruker EVOQ triple quadrupole gas chromatography-mass spectrometer (Bruker, MA, USA) in the authors’ laboratory (PMH) (left panel). The instrument is used to provide metabolic profiling for the diagnosis of inborn errors of metabolism by providing semi-quantitative information on urinary metabolites such as simple sugars, amino acids, dicarboxylic acids, nucleotides and pterins. Behind the door of the column oven (A, left panel), the 15-m column is compactly coiled into a circular bundle (top right). The heated sample enters the column at inlet C and eventually emerges from the column through D, to reach the ion source (bottom right). The remarkable length of the chromatography column accounts for the separating power of the gas chromatograph.

GC-MS begins with gas chromatography, typically with a capillary column 15-30 meters in length (Fig. 8): it requires a sample which is either already in the gaseous state, as it is done with headspace techniques [235], or that the sample is volatile enough to be vaporized in the gas chromatograph, typically operated at temperatures of 200-300 degree Celsius [86]. Derivatization procedures which modifies active hydrogens (carboxylic acids, alcohols, sulfhydryl group, amino groups, and amide groups) and ketone groups in the analyte are employed such that analytes in the sample become sufficiently volatile and thermally stable to be analysed in GC-MS [236].

The most common type of derivatization used in the clinical laboratory is trimethylsilyl (TMS) derivatization. BSTFA [N,O-bis(trimethylsilyl) trifluoroacetamide] and MSTFA [N-methyl-N-trimethylsilylfluoroacetamide] are the more common derivatization agents used. TMS derivatization result in the conversion of carboxylic acid (-COOH), hydroxyl (-OH), amino (-NH) and sulfhydryl (-SH) functional groups into their respective TMS derivatives [[236], [237], [238]]. In our laboratory, the combination of BSTFA+TMCS (99:1) is used as BSTFA reacts more rapidly than BSA and TMCS [trimethylchlorosilane] enhances the reactivity of BSTFA [237]. On the other hand, if measurement of ketone-group containing compounds is desired, oxime derivatization can be done. This is done by first derivatizing with methyl-oxime followed by TMS derivatization [230]. Some compounds which are measured in the clinical laboratory do not form stable TMS derivatives, for example barbiturates. These would require other derivatization methods such as alkylation with trimethylanilinium hydroxide (TMAH) [239,240].

If the target compound is volatile, however, instead of liquid injection, a technique known as head-space gas chromatography can be used where the mixture is heated in the vial and the vapor in the liquid is sampled and injected for analysis [235].

GC-MS can be operated in either scan mode or selected ion monitoring (SIM) mode. In scan mode, the quadrupole operates to scan for each mass the in the mass range specified, whereas in SIM mode, only specific ions are monitored. The scan mode allows a broad spectrum, untargeted profiling of a sample, whereas in SIM mode, specific analytes are measured and it offers much higher sensitivity.

The most common use of GC-MS analysers in a clinical chemistry laboratory is for detection of organic acids which can be used to diagnose a variety of inborn errors of metabolism such as disorders of the urea cycle, fatty acids oxidation disorders, and organic acidurias. GC-MS based organic acid detection has been described more than 40 years ago [224,241,242]. Testing for urine organic acids generally involves the extraction of organic acids by acidification followed by ethyl acetate extraction and TMS derivatization. Subsequent publication showed differing use of analytical columns, the use of SIM versus scan mode, and whether oximation of the specimen has been done [233]. The pros and cons of SIM versus scan mode are well illustrated in their use in urine metabolic screening. The use of SIM mode provides increased sensitivity; though the increased sensitivity may not be extremely significant as most metabolites monitored in urine metabolic screening is usually in high μmol/L levels, such sensitivity gained may translate into smaller injection volume, with resultant decrease in the need for equipment maintenance.

On the other hand, the use of scan mode provides flexibility; integration of peaks in the total ion chromatogram combined with library search using industrial-standard EI libraries allows identification of abnormal metabolites even though it is not on the list of monitored compounds (Fig. 1). Such procedures, however, cannot replace the integration of selected ions for critical analytes with lower concentrations, as they may co-elute with other organic acids which will lead to a false negative result.

#### 3.3.1 Metabolomics in the Clinical Laboratory

In the authors’ laboratory, urine metabolic screening is performed by drying of urine samples followed by derivatization by BSTFA/TMCS which enables simultaneous detection of amino acids, purine and pyrimidines, as well as simple sugars in the urine specimen [243,244]. Data analysis is done by library searching integrated peaks and by obtaining extracted ion chromatograms for selected analytes.

The detection of amino acids in GC-MS is not as straight-forward as in LC-MS/MS due to two issues. First, amino acid derivatization is inconsistent: while the carboxylic acid group is consistently derivatized, this is not necessarily the case for the amino group [245]. Second, carbamoylated amino acids such as citrulline and homocitrulline are transformed into their respective decarbamoylated forms, such as ornithine [246] and lysine. However, the detection of amino acids avoids the need of MO-TMS derivatization of ketones as most important keto-acid metabolites in urine originates from amino acids, the detection of which obviates the need for their detection.

GC-MS is also used for selective quantitation of very long chain fatty acids, cholestanol, phytosterols (e.g. sitosterol, stigmasterol), gamma-hydroxybutyric acid, and cyanide and its metabolites (thiocyanate; ATCA [2-aminothiazoline-4-carboxylic acid]), broad spectrum toxicology screening, targeted analysis of pesticides, and urine steroid profile [230].

### 3.4 Automation in Clinical Mass Spectrometry

Automation as applied to mass spectrometry-based assays involves automation in the management of specimens, sample preparation, the analytical run, data processing, and automated uploading of data to a laboratory information management system (LIMS) [247]. In the past decade, there have been dramatic advances in the automation of clinical mass spectrometry through automated sample preparation and the ability to interface mass spectrometers with LIMS.

Sample preparation techniques are employed most commonly to concentrate the analyte of interest, and to remove potential interfering components of the matrix to enhance detection [248]. Sample preparation may also involve derivatization, and in vitro generation of the measurand: an example being quantitation of the serum enzyme renin by its activity [249], commonly used in clinical endocrinology for the investigation of hypertension [250]. In the clinical laboratory, the choice is often between using general purpose liquid handling systems [251] and purpose-built sample preparation units, either standalone units [252] or integrated “on-line” into the chromatography/mass spectrometry suite. In the authors’ laboratory, on-line solid phase extraction is used in the determination of urine fractionated metanephrines for the diagnosis of phaeochromocytomas and paragangliomas and targeted analysis of drugs of abuse [253].

Connectivity of mass spectrometer suites to the LIMS in the clinical laboratory enables processed data to enter the hospital database accessible from the clinical management system without transcription by the operator which saves operator time as well as reduce transcription error, and enables central review of quality control data with other analysers in the laboratory [254].

### 3.5 Mass Spectrometry Bioinformatics in the Clinical Laboratory

With the advance in mass spectrometry, the modern high resolution mass spectrometer is able to acquire mass spectra at an extremely high rate with great sensitivity. This has been applied in the field of proteomics as digital biobanking of proteomes [255]. In contrast to the field of proteomics and metabolomics in which the use of open source software as well as open standards for mass spectrometry data has been widespread [[256], [257], [258], [259]], the use of mass spectrometry in clinical laboratory remained mostly within the framework of manufacturer-provided software as most applications of mass spectrometry in the clinical laboratory calls for selective quantitation of analytes rather than omic-profiling and data-mining. One starting point for a beginner in mass spectrometry data analysis is bioconductor (http://bioconductor.org/), an open source platform for bioinformatics data analysis [260,261].

An untargeted acquisition technique with LC-TOF instruments has been applied in the field of toxicology to enable re-analysis data as new psychoactive substances become known to the laboratory [262]. This comes, however, at the cost of data storage and the complexity of data analysis: a short 7-minute toxicology analytical run with a modern LC-QTOF analyser operating in full scan mode produces a file over 500 MB in size. Coupled with accreditation requirement of clinical laboratory this could require significant investment in digital storage equipment in the laboratory: at the time of writing, the local clinical laboratory accreditation body that oversees the laboratories of the authors require storage of analyser data for 3 years (The Hong Kong Laboratory Accreditation Scheme, Hong Kong Accreditation Service, https://www.itc.gov.hk/en/quality/hkas/hoklas/about.htm) which amounts to more than 12 terabytes of data for a laboratory with 30 specimens a day. It is therefore of great importance for the clinical laboratory to consider data storage solutions when acquiring a new high-resolution mass spectrometer.

### 3.6 MS Avec Frontiers: The Limitations of MS and How to Solve Them

While mass spectrometry is a promising and powerful technique, it should not be considered a panacea for quantitative measurement in the clinical laboratory. Matrix effect and isobaric interference are traditionally the two dominant categorical difficulties in mass spectrometry [263]. A third emerging category is that associated with protein measurement, as can be seen below.

#### 3.6.1 Matrix Effect

Matrix effect in mass spectrometry refers to the impact of non-analyte constituents in the sample on the ionization of the analytes. It is termed ion suppression when the effect is to decrease the ionization efficiency, and vice-versa, ion enhancement [264]. Due to the presence of endogenous components in clinical samples, ionization suppression or enhancement may occur for bioanalytical assays using LC-MS or LC-MS/MS technologies. The matrix effect may affect the precision and accuracy of a bioanalytical method and, therefore, compromise the quality of the results. Assay interference from the matrix should be considered when a new MS-based method is being developed: cleaner sample preparations, more sensitive instruments (allowing a smaller amount of, or a diluted to be analysed) and optimizing chromatographic separations for the analyte of interest [265].

Matrix effect is strongly dependent on the ionization techniques used, for example, it is more significant in ESI than in APCI [263], and in LC-MS than in GC-MS [266]. Matrix effect is contributed by the characteristics of both specimen and solvent used during the analysis: the ion suppression and enhancement effects are due to presence of other analytes that competes for ionization, provides alternative routes of ionization [263], suppresses crucial steps in ion formation in the ion source and binding to or reacting with the analytes [267]. Logically, it follows that the less separation there is, the more prone is an analytical technique to matrix effects. Elaborate extraction methods as used in reference measurement procedures [268] or definitive techniques [227] virtually eliminate co-eluting substances, but are often impractical in the clinical laboratory. As such, the focus of developing a routine method used in the clinical laboratory is to identify, reduce, and appropriately compensate for matrix effects and to caution the clinical user against such.

Identification of matrix effect can be done through post-column infusion experiment [269]. The post-column infusion experiment is done by mixing (1) the post-column eluent of blank matrix (an analyte-free specimen of the same sample type e.g. drug-free serum) and (2) standard solution through a T-piece before mass spectrometric detection and monitoring the change of signal over time [267]. Reduction is effected through extraction, clean-up, and separation [[270], [271], [272]], by using sample preparation techniques which range from liquid-liquid extraction to immunoaffinity solid-phase extraction, and through chromatography. Compensation is done by the use of appropriate internal standards (below), either isotopically labelled or structurally similar, the use of matrix-matched calibrators [273], or simply sample dilution [274].

Internal standards are chemicals that are added to the specimen prior to sample preparation in order to identify and correct for analytical variability from sources such as extraction recovery [275]. A stable isotope-labelled internal standard is an internal standard which is modified from the analyte of interest by replacing some of the atoms with their stable isotopes: D (deuterium), ^13^C, ^15^N, and less commonly, ^17^O. Stable isotope-labelled internal standards had near-identical chemical properties when compared with the analyte of interest, and would often elute at the same retention time as the analyte of interest and can therefore compensate for ion suppression/enhancement [276].

Stable isotope-labelled internal standard should be chosen carefully. Firstly, they must be adequately labelled such that it does not overlap with the natural isotope patterns of the analyte [277]. Difficulties may arise with isotope-labelled internal standards with multiple deuterium labels as they may be partially resolved from the analyte in chromatography [270]. To complicate the issue, deuterated internal standards of some endogenous metabolites and novel drugs may not be commercially available or may require dedicated synthesis and is therefore very expensive. Isotopically labelled internal standard could only compensate for the concentration determined: in the face of ion suppression, the signal is lowered and the resultant increase in uncertainty of measurement cannot be compensated [278].

#### 3.6.2 Isobaric Interference

Isobaric interference refers to the detection of ions of the same mass-to-charge ratio. These can result from structural and optical isomers, as well as multiple-charged ions [263]. Interferences from structural isomers are less important when mass spectrometry is combined with chromatography [279] but can represent a technical challenge in certain clinical applications, such as in new-born screening where only a single flow injection is done and leucine, isoleucine, and allo-isoleucine (structural isomers, all with a molecular formula C_6_H_13_NO_2_ and a molecular mass 131.1729) are being measured together [280]. On the other hand, optical isomers may be more difficult to separate, as it is seen with epitestosterone in the earlier years [281] and with the 3-epimer of 25-hydroxyvitamin D analyte [282]. This interference is notable because this 3-epimer typically does not interfere in immunoassays or competitive protein binding assays that are traditionally used for 25-hydroxyvitamin D measurement [283,284].

Paradoxically, isobaric interference may arise from the labelled internal standard, which itself is a powerful tool to combat other interferences: if there is inadequate labelling of the internal standard, isotopic interference may occur [285]. Furthermore, even if the labelled internal standard is adequately deuterated, exchange of deuterium from the internal standard may still occur [286]. This highlights the balance between adequate deuteration of internal standard to prevent cross-talk by high levels of analytes, and the different elution times [287]. Isobaric interference also occurs as a matter of routine in ICP-MS measurement and mathematical approaches are used, for example, correction of measurement of ^58^Ni from ^58^Fe by calculation with the ^57^Fe measurement [288].

The strategy to eliminate isobaric interference depends on the nature of interference. As applied to the interferences of 3-epimer of 25-hydroxyvitamin D and epitestosterone, chromatography separation would abate the interference and may even allow for determination of their concentrations [283,289]. Analytes with different elemental composition but same nominal mass can be distinguished by high resolution mass spectrometry techniques such as TOF-MS [290], and TOF-MS techniques have also been applied to elemental analysis by ICP-MS [291] which obviates the need of mathematical calculation. Each of these strategies, however, has its own limitations: better separation techniques add to the analytical time [283], and high resolution mass spectrometry are more expensive than their unit-resolution counterparts, and had lesser dynamic range [292].

#### 3.6.3 Protein Molecular Variants

Quantitative measurement of proteins with mass spectrometry involves either measurement of the intact protein as a multi-charged molecular ion [293] or peptides obtained from digestion by proteolytic enzymes such as trypsin [294]. Molecular variants in the human genome is well described [295], and large population-derived databases of proteomic [296] and genomic [297] variants are available for searching. The implication of molecular variants is that change in amino acids would result in changes in the molecular mass, and hence the mass-to-charge ratio of the analyte of interest; this has resulted in erroneous measurement in the measurement of IGF-1 due to presence of the A70T amino acid amino acid variant [298]. This highlights the need for the consideration of single nucleotide polymorphisms and their respective allele frequencies in designing quantitative protein assays using mass spectrometry for detection [299]. Strategic planning of an clinical MS-based assay will not only avoid these pitfalls, but should also allow the assay to surpass the performance of traditional immunoassays due to the simultaneous discrimination and quantification of the clinically relevant sequence variations [300].

#### 3.6.4 Is Mass Spectrometry the Right Tool for the Job?

Automation of mass spectrometric analysis is still in its preliminary stage [301], and while sample preparation techniques like online solid phase extraction are available [302], the majority of clinical laboratories still employ significant amount of manual techniques in sample preparation. Henceforth round-the-clock mass spectrometry services, though reported in the literature [303], remained a rarity among clinical laboratories, at least in the authors’ experience. Another question to consider before implementation of a mass spectrometry technique is whether mass spectrometer, as an alternative to, for example, a chromatographic detector, is the appropriate tool for the job: other detectors with GC and LC such as diode array detector and nitrogen thermionic specific detectors are less expensive and require less expertise, and do not necessitate isotopic internal standards [304].

## 4 Conclusion

In the past decades, we have seen revolutionary changes in the clinical laboratory: from advanced automation and laboratory informatics systems, widespread use of molecular techniques and most recently, MS-based methods. From the vantage point of time, this sequence of events seems, at least retrospectively, logical and well-anticipated. Invention of immunoassay fuelled the surge of automatic analysers. Breakthroughs in engineering and computer science accumulated in the genetic era eventually led us to the genomic age. While it is beyond doubt that the importance of MS-based techniques in clinical laboratories will continue to grow, we cannot predict its growth trajectory with certainty. Will MS-based techniques merge with the next-generation DNA sequencing techniques to give rise to efficient and economical sequencing solutions applicable to the service laboratory [305,306]? Will MS-based techniques attain an adequate level of automation and ease-of-operation to replace majority of the chemical and immunological analysers in the clinical laboratory [173]? Will MS-based techniques see breakthroughs in miniaturization which allow them to move, literally, *from bench to bedside*, to serve as point-of-care testing devices [307]?

Due mainly to the vast scope of the topic, in this mini-review, we have selected and outlined only practical examples and technologies that are pertinent to our practice and are fully mature for adoption in clinical laboratories with compatible resources. We did not include assays that are usually limited to reference laboratories, such as certain mycotoxin assays [308] and cerebrospinal fluid neurotransmitter profiling methods [309,310]. The research applications of MS-based techniques, for example in elucidating novel disease markers [311], microbial metabolomic profiles [[312], [313], [314], [315], [316]], identification of novel toxins [[317], [318], [319], [320]] and characterizing chemical contaminations of public health importance [321,322], have also been excluded. We should stay vigilant, not only because MS-based techniques have the potential to replace current techniques and existing expertise and instrument may undergo rapid evolution, but also because the emergence of this technique is linked intricately to generation of massive data – similar to the early days of next-generation DNA sequencing. Change-embracing yet analytical minds are required to unleash the full potential of this rapid-dominating platform and at the same time establish quality assurance measures to insure accuracy of laboratory results and patient safety.

We decide to end our mini-review with the all-too-often quoted from the wartime British Prime Minister:

*“Now this is not the end. It is not even the beginning of the end. But it is, perhaps, the end of the beginning.”* – Winston Churchill

(1874 - 1965)

This is certainly true for Mass Spectrometry in the Clinical Laboratory.
